# Opportunistic Screening for Acute Vertebral Fractures on a Routine Abdominal or Chest Computed Tomography Scans Using an Automated Deep Learning Model

**DOI:** 10.3390/diagnostics14070781

**Published:** 2024-04-08

**Authors:** Ye Rin Kim, Yu Sung Yoon, Jang Gyu Cha

**Affiliations:** 1 Department of Radiology, College of Medicine, Soonchunhyang University Bucheon Hospital, Soonchunhyang University, Bucheon 14584, Republic of Korea; 2 Department of Radiology, School of Medicine, Kyungpook National University Hospital, Kyungpook National University, Daegu 41944, Republic of Korea

**Keywords:** deep learning, artificial intelligence, vertebral compression fracture, spine, computed tomography

## Abstract

Objectives: To develop an opportunistic screening model based on a deep learning algorithm to detect recent vertebral fractures in abdominal or chest CTs. Materials and Methods: A total of 1309 coronal reformatted images (504 with a recent fracture from 119 patients, and 805 without fracture from 115 patients), from torso CTs, performed from September 2018 to April 2022, on patients who also had a spine MRI within two months, were included. Two readers participated in image selection and manually labeled the fractured segment on each selected image with Neuro-T (version 2.3.3; Neurocle Inc.) software. We split the images randomly into the training and internal test set (labeled: unlabeled = 480:700) and the secondary interval validation set (24:105). For the observer study, three radiologists reviewed the CT images in the external test set with and without deep learning assistance and scored the likelihood of an acute fracture in each image independently. Results: For the training and internal test sets, the AI achieved a 99.86% test accuracy, 91.22% precision, and 89.18% F1 score for detection of recent fracture. Then, in the secondary internal validation set, it achieved 99.90%, 74.93%, and 78.30%, respectively. In the observer study, with the assistance of the deep learning algorithm, a significant improvement was observed in the radiology resident’s accuracy, from 92.79% to 98.2% (*p* = 0.04). Conclusion: The model showed a high level of accuracy in the test set and also the internal validation set. If this algorithm is applied opportunistically to daily torso CT evaluation, it will be helpful for the early detection of fractures that require treatment.

## 1. Introduction

Vertebral compression fractures (VFs) are the most common osteoporotic fractures, and are associated with significant morbidity and mortality [1]. Early diagnosis is crucial to prevent further complications [1,2,3]. However, VFs can be challenging to discern clinically as they are often asymptomatic or have nonspecific symptoms [4]. Also, incidental VFs that are found during routine chest and abdominal computed tomography (CT) scans are often under-reported due to the lack of sagittal spine reconstructions and the limited awareness of abdominal radiologists about spines [5,6,7,8,9,10].

Some efforts have been made to use machine learning to aid in the detection of spinal fractures in various radiology exams [11,12,13,14,15,16,17,18,19], and also to further differentiation between benign and malignant fractures [17]. Only two of them used CT scans [11,12], but sagittal reconstruction images, which are not routinely included in opportunistic torso CT scans, were used, and both acute and old fractures, which were of less clinical significance, were included [20].

So, the purpose of this study was to (1) develop a deep learning model for the automatic detection of particularly acute vertebral compression fractures on routine chest and abdominal CT scans, (2) evaluate the performance of this model in automatically detecting incidental acute VFs, and (3) compare the performances of observer with and without the model’s assistance.

## 2. Materials and Methods

**Figure 1:**
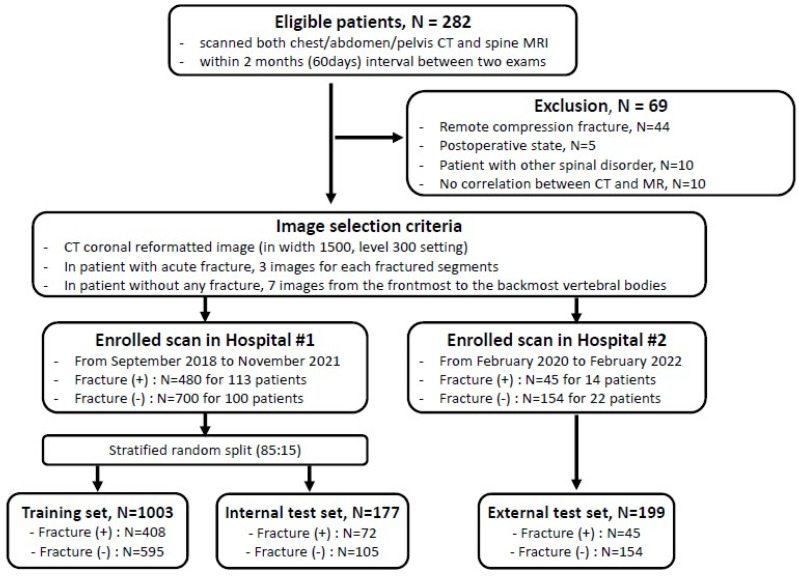
Flow chart of selecting the study sample.

An overview of the datasets is shown in Figure 1. This retrospective study complied with the principles of the Helsinki Declaration and was approved by the institutional review board (IRB) of two participating hospitals: Hospital #1 (Soonchunhyang University Bucheon Hospital) for the internal test set and Hospital #2 (Soonchunhyang University Cheonan Hospital) for the external test set, respectively.

### 2.1. Patient Datasets

Among the patients who underwent both (1) chest or abdominal CT and (2) MRI of the thoracic or lumbar spine, corresponding to the CT scan’s range, patients with less than two months between the two exams were included. An acute VF was identified on the spine MRI, and if the lesion correlated to the CT image, it was classified as being in the fracture-positive group. On the other hand, patients without any fractures in either CT or spine MRI were classified being in the fracture-negative group. Patients who were in a post-operative state following any kind of spinal surgery, such as metallic hardware or bone cement insertion, or with any other pathology including bone tumors (such as bone metastasis from other malignancy), the involvement of bone marrow disease (such as multiple myeloma), or infectious spinal conditions were excluded from the study. Finally, we included 113 fracture-positive and 100 fracture-negative patients identified from September 2018 to November 2021 in Hospital #1 and 14 fracture-positive and 22 fracture-negative patients identified from February 2020 to February 2022 in Hospital #2, respectively.

### 2.2. Image Selection

All CT images were scanned using a soft-tissue reconstruction kernel with a 3 mm slice thickness and without an interval gap across five different CT scanners. All selected images were saved in bone setting (1500 width and 300 level) from the picture archiving and communication system (PACS). One radiologic resident (with 2 years of in-house training, reader (1) and one board-certified musculoskeletal radiologist (with 8 years of experience, reader (2) selected three serial coronal-reformatted images with 3 mm gaps from the front which showed well-visible fracture lines for each of the fractured segments in the fracture-positive group of patients and seven serial coronal-reformatted images with 6 mm gaps from the frontmost vertebral body for each of the patients in the fracture-negative group. In all, a total of 480 and 700 images from the fracture-positive and -negative groups in Hospital #1, respectively, were used as the development dataset. Similarly, in Hospital #2, 45 and 154 images from the fracture-positive and -negative groups, respectively, were selected for use as the external test set by another board-certified musculoskeletal radiologist (with 5 years of experience, reader 4).

### 2.3. Deep Learning Model Development

**Figure 2:**
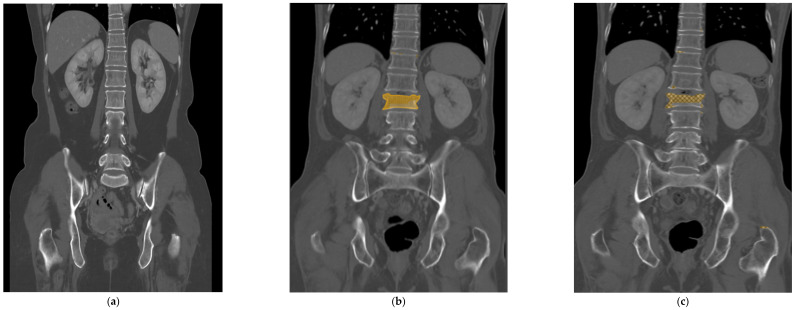
The selected input image was resized to 512 × 512 pixels (**a**). Using the Neuro-T software, version 3.0.0 architecture (Nerocle Inc., Seoul, Republic of Korea), a yellow-colored polygonal box was drawn manually along the outer margin of the cortex, which had the fracture confirmed on a recent MRI, including as many bone fragments as possible (**b**). After the deep learning process was trained on these features, the predicted fractured areas, for which predicted scores ranged from 50 to 100%, were shown on the image with a checked pattern in pixels (**c**). This case was evaluated as a true positive result.

**Figure 3:**
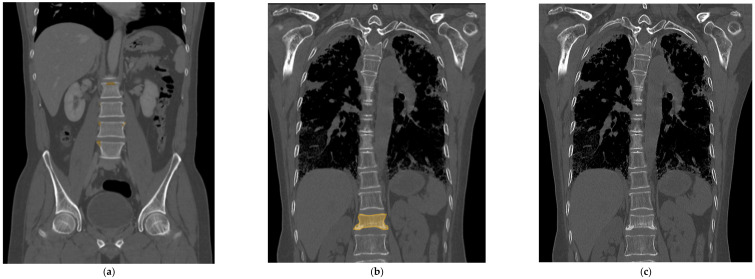
As a case of false positives, the trained deep learning model color-mapped areas suspected of having fractures, but in reality, these did not have any fractures (**a**). However, these false positive results have a tendency to be found in the high attenuated cortex showing marginal osteophytes of the vertebra or normal endplates. In this case of a false negative, the fractured vertebra segment confirmed on the MRI was colored and trained (**b**), but the deep learning model could not recall a fractured segment when there was no checkered pixel (**c**). It appeared only as a subtle and narrow condensation zone on the CT, making it challenging to suspect a fracture even on the actual raw CT image.

All the selected images were resized to 512 × 512 pixels and uploaded to Neuro-T, version 3.0.0 architecture (Neurocle Inc., Seoul, Republic of Korea). Then, as shown in Figure 2, any visibly fractured vertebral bodies in each image were labeled manually with a colored polygon along the outer margin of the cortex, including as many bone fragments as possible identified by one radiologist (reader 2). The dataset was randomly divided into 85% training and 15% test datasets for model development and accuracy predictions. The primary outcomes of the model were the test accuracy, precision, and F1 score. After training the model using the images, the predicted score of predicted fractured areas was calculated, ranging from 50 to 100%, and displayed as boxes and pixels on the image, like in Figure 2 and Figure 3. The predicted scores then were evenly classified into four groups (no score, normal; from 50 to 66.67%, indeterminate; from 66.68 to 83.33%, probable fracture; above 83.34%, fracture).

### 2.4. Observer Study

For the observer study, three radiologists (reader 1, 2, and a board-certified musculoskeletal radiologists with 23 years of experience, reader 3) participated in the review of the CT images in the external test set, to which patients were randomly allocated, and all patients’ information was anonymized. All readers independently scored the likelihood of acute VF in each image on a 4-point scale ((1), normal; (2), probably normal; (3), probable fracture; (4), definite fracture). After more than 1 week, we performed the experiment in the same way with the support of a deep learning model, and used highlighted boxes to show the AI-predicted fracture regions on each image and the AI-predicted scores, while knowing the previous scores.

### 2.5. Statistical Analysis

The statistical analyses were performed using Rex version 3.0.3 (RexSoft, Seoul, Republic of Korea). Continuous data for patients’ age and the period between the CT and MRI exams were presented as means and standard deviation, whereas categoric data were presented as counts and percentages. To evaluate the diagnostic performance of the groups, a 4-point scale was dichotomized into normal, 1 and 2 points, and fracture, 3 and 4 points, for binary diagnosis. Then, the performance outcomes were derived from an area under the receiver operating characteristic curve (AUROC) for each human reader, with and without AI assistance, and also for the stand-alone AI reader. A comparison of individual AUROC values was carried out using Delong’s method. Sensitivity, specificity, accuracy, and error rate values for each reader were also calculated and compared using McNemar’s test, and positive predictive values (PPVs) and negative predictive values (NPVs) were calculated using the Chi-square test. A *p* value of less than 0.05 was considered to be statistically significant.

## 3. Results

### 3.1. Patient Characteristics

Of the 213 patients in the internal test set, 113 had acute VFs (mean age, 61.2 years ± 19.5 [standard deviation, SD]; 60 male) and 100 had no fracture (mean age, 56.1 years ± 14.6 [SD]; 57 male). Among the 36 patients in the external test set, 14 had acute VFs (mean age, 73.9 years ± 12.6 [SD]; 8 male) and 22 had no fracture (mean age, 61.2 years ± 18.0 [SD]; 9 male). A total of 160 and 15 fractured segments were found in 113 and 14 patients from the internal and external test sets, respectively, and a total of 480 and 45 CT images were included with three images per segment (Figure 1). Almost all fractures occurred at the thoracolumbar junction and in the lumbar spine, from T11 to L5 (98.1% and 93.3% for internal and external test sets, respectively), and others occurred at the T6, T8, and T10 vertebrae. The characteristics of the patients in the datasets are listed in Table 1.

### 3.2. Stand-Alone AI Performance

The trained deep learning model achieved a 99.89% accuracy, 92.00% precision, and 92.40% F1 score in the internal test set, respectively. The total training time was approximately 13 h. When retesting with the external test set, the model was able to reach a 99.91% accuracy, 80.30% precision, and 85.50% F1 score, respectively. It took 30 min to test the set with the trained model. Also, in the external test set, the model showed an AUROC of 0.9889 (95% confidence interval [CI], 0.9762–0.9977), a sensitivity of 86.67% (95% CI, 73.21–94.95), a specificity of 100% (95% CI, 94.56–100), a PPV of 100% (95% CI, 90.97–100), and an NPV of 91.67% (95% CI, 82.74–96.88) (Table 2). Figure 3 shows one of the cases of false-positive and false-negative results, respectively, from the internal test set.

### 3.3. Observer Study

**Figure 4:**
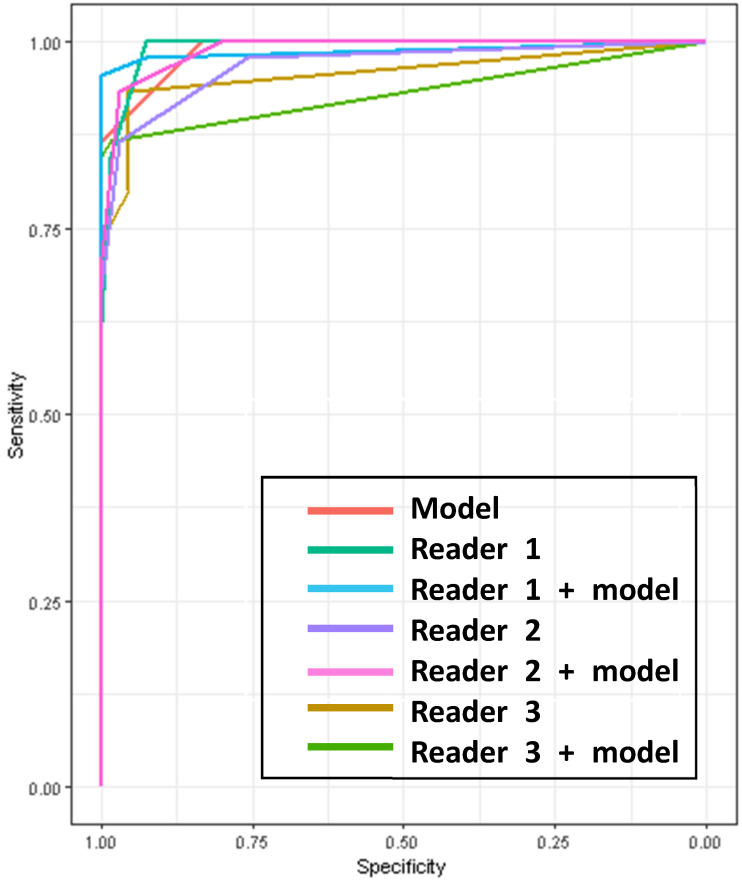
AUROC curves of the model and the readers for diagnosis of vertebral fractures on the external test set. AUROC = area under the receiver operating characteristic curve.

The diagnostic performances of the human observers with and without the model’s assistance in the external test set are shown in Table 2 and Figure 4. In the setting without the model’s assistance, the AUROCs of the readers, including AI alone, ranged from 0.9576 to 0.9912 and there were no significant differences between all the readers (Table 3).

With the model’s support, only one category, the accuracy of reader 1, who was the radiologic resident, was improved significantly from 92.79 (95% CI, 86.29–96.84) to 98.2 (95% CI, 93.64–99.78) (*p* = 0.04) (Table 2). The AI did not provide significant assistance to the AUROCs of the diagnostic performances of each reader (Table 3, Figure 4). But the AUROC of reader 3 with AI assistance (0.9322, [95% CI, 0.8871–0.9768]) showed a statistically significantly lower value compared to the other AI-assisted readers (AI-assisted reader 1, 0.9872, [95% CI, 0.9637–1], *p* = 0.02, and AI-assisted reader 2, 0.9897, [95% CI, 0.9777–0.996], *p* = *0*.01)

## 4. Discussion

As the population ages, complications from osteoporosis, such as vertebral fractures, are becoming more common. However, these vertebral body compression fractures are often underdiagnosed in abdomen CT scans, and there are some concerns about this. Previous studies have explained that this is because many patients are asymptomatic, there is often a lack of sagittal images, and abdominal radiologists often focus only on the solid organs in the abdomen, making it easy to overlook the bony structures [6,9,10]. In previous studies, machine learning systems have been developed to automatically detect such vertebral compression fractures with high levels of sensitivity, accuracy, and precision. They have used plain spine radiography [13], spine CT [14], and chest, abdomen, and pelvis CT scans [12]. However, even with visceral CT scans available, Tomita N et al. investigated the use of sagittal CT scans, which are not routinely included, and focused only on the presence or absence of fractures, without distinguishing between the acute and remote stages. As far as we know, this is the first time that an acute fracture has been used to automatically detect compression fractures on an abdomen or chest CT using a deep learning model.

The results from our deep learning model showed higher performance compared to previous studies with an accuracy of 99.89% and a precision of 92.00% during internal validation, and an accuracy of 99.91% and a precision of 80.30% during the external test set, respectively. When reviewing the true positive cases, the deep learning model accurately filled more than 75% of the fractured area in the fractured vertebral body square with colored mapping. On the other hand, false-positive cases were only shown in a minimal part of the vertebral body, mostly at marginal osteophytes or normal endplate areas. Therefore, the readers could easily distinguish false positives by simply looking at the colored area. Only one false negative was seen, which was a case where the condensation zone was very subtle and narrow and was difficult to detect even on the raw CT image.

In the observer study, a significant improvement in performance was only seen in the resident’s accuracy with the AI’s assistance, being 98.2 compared to 92.79, with a *p*-value of 0.0412. The other results did not show a significant improvement when the AI was incorporated. No significant difference was seen in the AUROC between the standalone reader, the AI-assisted reader, or AI alone. This suggests that there is utility in AI, whether used alone or as an assistant. Additionally, the performance of the AI can be considered equal to that of a practicing radiologist.

In addition, it is known that sagittal scans are useful for diagnosing compression fractures [21,22], but it has also been found that high diagnostic rates can be obtained even when only using coronal images, which are almost always included in routine CT scans, without the need for additional reconstruction efforts. This indicates that coronal images alone can be used to diagnose the fracture adequately.

This was a retrospective study from a single institution, and therefore there was a limitation associated with modelling the data due to the sample size. However, the study included images taken with different CT machines at a single institution, and also, for external validation, included images taken using different CT machines and protocols at another institution, which may help to address this limitation. Further studies with larger cohorts across multiple institutions are also needed.

The patient population in this study consisted of those who underwent CT scans and had a recent vertebral fracture diagnosed within two months using a spine MRI, so it was not representative of all vertebral fractures.

Also, the deep learning model was unable to detect remote fractures and analyze the results, so it was not possible to evaluate how well it could differentiate acute fractures from remote lesions. However, the study’s significance lies in the fact that it focused on detecting acute lesions that can cause the patient’s current symptoms and require additional treatment.

Another limitation is that the performance of musculoskeletal radiologists, and not abdominal radiologists who are expected to use the model in practice, was analyzed, and there was also a limited number of readers. However, the results of the study showed that the AI itself demonstrated a performance that was at the same level as the trained musculoskeletal radiologists, which means that it can be a great help not only to non-specialists, including abdominal radiologists, but also to clinical physicians.

The automatic segmentation of the vertebral body has not yet been implemented due to the technical limitations of the software. Also, despite the differences in background bone density based on age and gender of the patients, these patient-specific co-factors cannot be analyzed due to these technical limitations. Future development of the software is necessary in this regard.

In this study, the trained automated deep learning model showed a high level of accuracy on both internal and external validation sets. Additionally, there was no significant difference in diagnostic performance between the fractures detected by the AI model and those detected by trained and less-trained radiologists. This could potentially reduce the workload of radiologists in detecting fractures, or even replace them. If this algorithm is applied in clinical practice, it can not only help in the early detection of acute vertebral compression fractures for patients but also help to reduce under-reporting by radiologists.

## Figures and Tables

**Table 1:** Characteristics of the patients and images.

Parameter	Internal Test Hospital		External Test Hospital	
	Without Fracture	With Fracture	Without Fracture	With Fracture
No. of patients	100	113	22	14
Age (years) *	56.1 ± 14.6	61.2 ± 19.5	61.2 ± 18.0	73.9 ± 12.6
No. of Men (%)	57/100 (57)	60/113 (53.1)	9/22 (40.9)	8/14 (57.1)
CT-MR scan interval (days) *	13.9 ± 16.0	8.0 ± 11.4	9.8 ± 11.4	4.2 ± 3.9
No. of CT scan-ordered department (%)				
- Orthopedic Surgery	22/100 (22)	26/113 (23.0)	0	0
- Neuro-Surgery	28/100 (28)	32/113 (28.3)	0	0
- Emergency Medicine	0	5/113 (4.4)	12/22 (54.5)	11/14 (78.6)
- Others	50/100 (50)	50/113 (44.2)	10/22 (45.5)	3/14 (21.4)
No. per fractured segment		Total 160		Total 15
- T11		6/160 (3.8)		0
- T12		32/160 (20)		3/15 (20)
- L1		42/160 (26.3)		4/15 (26.7)
- L2		36/160 (22.5)		5/15 (33.3)
- L3		20/160 (12.5)		1/15 (6.7)
- L4		18/160 (11.3)		0
- L5		3/160 (1.9)		1/15 (6.7)
- Others		3/160 (1.9) **		1/15 (6.7) ***

* Data are means ± standard deviations. ** others including T6, T8, and T10 vertebrae. *** the one involving T6 vertebra.

**Table 2:** Diagnostic performance of the AI model and three readers for acute fracture detection with and without AI assistance.

Total (*n* = 111)	AI	Reader 1			Reader 2		Reader 3	
		Without AI		With AI	Without AI	With AI	Without AI	With AI
AUROC	0.9889 (0.9762–0.9977)	0.9912 (0.977–0.999)		0.9872 (0.9637–1)	0.968 (0.9437–0.9937)	0.9897 (0.9777–0.996)	0.9576 (0.9142–0.9936)	0.9322 (0.8871–0.9768)
Sensitivity		84.44 (70.54–93.51)		95.56 (84.85–99.46)	86.67 (73.21–94.95)	93.33 (81.73–98.60)	80 (65.4–90.42)	86.67 (73.21–94.95)
: *p*-value		0.07			0.25		0.25	
Specificity	100 (94.56–100)	98.48 (97.84–99.96)		100 (94.56–100)	96.97 (89.48–99.63)	96.97 (89.48–99.63)	95.45 (87.29–99.05)	98.48 (91.84–99.96)
: *p*-value		1			NA		0.48	
Accuracy	94.59 (88.61–97.99)	92.79 (86.29–96.84)		98.2 (93.64–99.78)	92.79 (86.29–96.84)	95.5 (89.80–98.52)	89.19 (81.88–94.29)	93.69 (87.44–97.43)
: *p*-value		0.04			0.25		0.07	
PPV	100 (90.97–100)	97.44 (86.52–99.94)		100 (91.78–100)	95.12 (83.47–99.4)	95.45 (84.53–99.44)	92.31 (79.13–98.38)	97.5 (86.84–99.94)
: *p*-value		0.96			1		0.59	
NPV	91.67 (82.74–96.88)	90.28 (80.99–96.0)	97.06 (89.78–99.64)		91.43 (82.27–96.79)	95.52 (87.47–99.07)	87.5 (77.59–94.12)	91.55 (82.51–96.84)
: *p*-value		0.12			0.53		0.61	

Note—Numbers except for AUROC are presented as percentage with 95% confidence intervals in parentheses. AI = artificial intelligence. AUROC = area under the receiver operating characteristic curve. PPV = positive predictive value. NPV = negative predictive value. NA = not applicable.

**Table 3:** *p*-values for comparison between AUROCs of each reader’s diagnostic performances with and without AI assistance.

Readers	Reader 1		Reader 2		Reader 3	
	−AI	+AI	−AI	+AI	−AI	+AI
AI	0.72	0.90	0.27	0.98	0.10	0.03
Reader 1 − AI	-	0.74	0.09	0.70	0.10	0.02
Reader 1 + AI		-		0.87	0.07	0.02
Reader 2 − AI			-	0.08	0.45	0.14
Reader 2 + AI				-	0.03	0.01
Reader 3 − AI					-	0.12
Reader 3 + AI						-

## Data Availability

The datasets used and/or analyzed during the current study are available from the corresponding author on reasonable request.
